# Tackling Research Inefficiency in Degenerative Cervical Myelopathy: Illustrative Review

**DOI:** 10.2196/15922

**Published:** 2020-06-11

**Authors:** Danyal Zaman Khan, Muhammad Shuaib Khan, Mark RN Kotter, Benjamin Marshall Davies

**Affiliations:** 1 Division of Neurosurgery Department of Clinical Neurosciences University of Cambridge Cambridge United Kingdom; 2 Wellcome Trust and Medical Research Council Cambridge Stem Cell Institute Anne McLaren Laboratory University of Cambridge Cambridge United Kingdom

**Keywords:** cervical, myelopathy, spondylosis, spondylotic, stenosis, disc herniation, ossification posterior longitudinal ligament, systematic review, research inefficiency, imprecision

## Abstract

**Background:**

Degenerative cervical myelopathy (DCM) is widely accepted as the most common cause of adult myelopathy worldwide. Despite this, there is no specific term or diagnostic criteria in the International Classification of Diseases 11th Revision and no Medical Subject Headings (MeSH) or an equivalent in common literature databases. This makes searching the literature and thus conducting systematic reviews or meta-analyses imprecise and inefficient. Efficient research synthesis is integral to delivering evidence-based medicine and improving research efficiency.

**Objective:**

This study aimed to illustrate the difficulties encountered when attempting to carry out a comprehensive and accurate evidence search in the field of DCM by identifying the key sources of imprecision and quantifying their impact.

**Methods:**

To identify the key sources of imprecision and quantify their impact, an illustrative search strategy was developed using a validated DCM hedge combined with contemporary strategies used by authors in previous systematic reviews and meta-analyses. This strategy was applied to Medical Literature Analysis and Retrieval System Online (MEDLINE) and Excerpta Medica dataBASE (EMBASE) databases looking for relevant DCM systematic reviews and meta-analyses published within the last 5 years.

**Results:**

The MEDLINE via PubMed search strategy returned 24,166 results, refined to 534 papers after the application of inclusion and exclusion criteria. Of these, 32.96% (176/534) results were about DCM, and 18.16% (97/534) of these were DCM systematic reviews or meta-analyses. Non-DCM results were organized into imprecision categories (spinal: 268/534, 50.2%; nonspinal: 84/534, 15.5%; and nonhuman: 8/534, 1.5%). The largest categories were spinal cord injury (75/534, 13.67%), spinal neoplasms (44/534, 8.24%), infectious diseases of the spine and central nervous system (18/534, 3.37%), and other spinal levels (ie, thoracic, lumbar, and sacral; 18/534, 3.37%). Counterintuitively, the use of human and adult PubMed filters was found to exclude a large number of relevant articles. Searching a second database (EMBASE) added an extra 12 DCM systematic reviews or meta-analyses.

**Conclusions:**

DCM search strategies face significant imprecision, principally because of overlapping and heterogenous search terms, and inaccurate article indexing. Notably, commonly employed MEDLINE filters, human and adult, reduced search sensitivity, whereas the related articles function and the use of a second database (EMBASE) improved it. Development of a MeSH labeling and a standardized DCM definition would allow comprehensive and specific indexing of DCM literature. This is required to support a more efficient research synthesis.

## Introduction

### Background

Degenerative cervical myelopathy (DCM) arises when degenerative changes of spinal structures cause myelopathy of the cervical spinal cord [1]. These degenerative changes include spondylosis, disc prolapse, hypertrophy, calcification, and ossification of the posterior longitudinal ligament and ligamentum flavum [1]. Ultimately, this results in stenosis of the spinal canal leading to cord compression, mechanical stretch, repetitive microtrauma, and chronic reduction in cord blood flow [1,2]. A complex pathological cascade follows with neuroinflammation, demyelination, neurodegeneration, and gliosis, resulting in the clinical entity we know as myelopathy [1,3,4].

The prevalence of DCM has proven difficult to ascertain owing to the novel umbrella term, difficulty in diagnosis, and the relative paucity of data [1]. Nevertheless, it is widely accepted as the most common cause of adult myelopathy worldwide [5]. DCM is not only very prevalent but also quite disabling, with the quality of life scores (36-Item Short Form Health Survey) in patients with DCM being lower than those in patients with most other common conditions, with heart failure and sciatica being identified as the only 2 conditions with lower scores [6].

Despite this, there remains no specific term or diagnostic criteria in the International Classification of Diseases 11th Revision (ICD-11), which encompasses the related and often coexisting conditions covered under the DCM umbrella [7]. Similarly, there exists no Medical Subject Headings (MeSH) term for PubMed (or an equivalent grouping index term for other databases) for DCM or its constituent terms (Textbox 1) [7]. MeSH terms improve the precision and efficiency of literature searches [8]. The hierarchical structure arrangement of MeSH *trees* allows for narrower terms to fall under the MeSH term heading and for search engines to consider other terms as MeSH synonyms [9]. This has proven particularly useful in medical terms that follow umbrella structures [9], for example, the MeSH *Spinal Cord Injuries* encompasses terms such as *spinal cord transection, traumatic myelopathy*, and *spinal cord contusions* and includes useful subheadings such as *etiology*, *diagnosis*, and *surgery*. This structure is likely to be useful in DCM terminology, which encompasses terms such as *cervical spondylotic myelopathy* and *ossification of the posterior longitudinal ligament* [1]. The relative novelty of the term DCM does not entirely explain its lack of MeSH terms. In 2019, 421 new MeSH terms were added to the Medical Literature Analysis and Retrieval System Online (MEDLINE)/PubMed database, some of which are in reference to other equally novel terms, such as emerging monoclonal antibody-based therapies and small molecule inhibitors [10]. Indeed, the use of free-text searches alone of DCM returns imprecision in the form of overlapping terms, including the subjects of noncervical myelopathy, noncervical spine degenerative changes, and gynecological cervix [7].

The lack of a consistent index term and MeSH term for DCM makes searching the literature imprecise and inefficient. This is particularly crucial when considering the importance of thorough systematic reviews and meta-analyses in reducing research wastage [11]. Indeed, in 2010, an estimated US $240 billion was spent on biomedical research, with an estimated 85% of this research wasted, resulting in no clinical translation or benefit [11,12]. Reviews of the literature increase research efficiency by preventing research duplication and directing future primary research [11]. However, previous surveys have indicated that over half of the clinical trial designers may be unaware of all the existing major reviews relevant to their study design [13]. The omission of this crucial step in informing trials has led to countless numbers of trials with inappropriate design, with one series highlighting that up to 75% of trials without the mention of systematic reviews or meta-analyses informing their protocols had trial designs that were considered inadequate [14,15]. In addition, systematic reviews and meta-analyses are important in preventing duplication of existing knowledge and in putting the results of trials into the context of existing literature so that the clinical relevance of findings is more interpretable [15].

**PubMed/Medical Literature Analysis and Retrieval System Online Medical Subject Headings (MeSH) terms contained within the Spinal Diseases and Spinal Cord Diseases categories. Of note, the Ossification of the Posterior Longitudinal Ligament (OPLL) MeSH that currently exists does not specify OPLL with radiculomyelopathy or OPLL without radiculomyelopathy.**

**Spinal diseases**

- Intervertebral disc degeneration
- Intervertebral disc displacement
- Ossification of the posterior longitudinal
- Platybasia
- Posterior cervical sympathetic syndrome
- Spinal curvatures
- Spinal neoplasms
- Spinal osteochondrosis
- Spinal osteophytosis
- Spinal stenosis
- Spondylitis
- Spondylosis
- Spondylolysis

**Spinal cord diseases**

- Pneumorrhachis
- Spinal cord compression
- Spinal cord injuries
  Central cord syndrome
- Spinal cord neoplasms
- Spinal cord vascular diseases
- Spinocerebellar degeneration
- Stiff-Person syndrome
- Subacute combined degeneration
- Syringomyelia
- Tabes dorsalis
- Amyotrophic lateral sclerosis
- Epidural abscess
- Spinal muscular atrophy
- Myelitis

### Objectives

Currently, the process of conducting systematic reviews and meta-analyses in many fields is laborious and inefficient [15,16]. This study aimed to illustrate the difficulties encountered when attempting to carry out a comprehensive and accurate evidence search in the field of DCM by identifying the key sources of imprecision and quantifying their impact.

## Methods

### Developing an Illustrative Search Strategy

Studies concerning DCM within the last 5 years were initially identified using a search filter/hedge, which has been previously validated for DCM and has returned a 100% sensitivity in DCM datasets [7]. Search strategies used in these systematic reviews and meta-analyses were compared with the search strategies of the validated hedge. The validated strategy was combined with the strategies that have been actively used by authors, forming our example search strategy (Textbox 2). Effectively, this resulted in the addition of the terms *Degenerative Cervical Myelopathy* and *Ossification of Posterior Longitudinal Ligament* to the strategy. This approach, rather than the exclusive use of the search filter, was chosen because it most closely aligned with the current search practices in DCM.

#### Inclusion and Exclusion Criteria

A search filter (Textbox 3) encompassing the inclusion and exclusion criteria was applied to our search terms. Relevant studies were identified through hand searching all articles returned after the application of the search filter.

**Search terms used to identify relevant studies.**

OR

- DCM/Degenerative Cervical Myelopathy
- OPLL/Ossification of Posterior Longitudinal Ligament
- CSM/Cervical Spondylotic Myelopathy
- JOA/Japanese Orthopaedic Association
- Cervical Vertebrae OR Cervical Cord

AND

- Myelopathy OR Myeloradiculopathy OR Spondylomyelopathy OR Spinal Cord Diseases OR Spinal Cord Disorder OR Spinal Cord Compression

**Search filters (Phase 1 and Phase 2) applied to the search terms.**

**Inclusion criteria**

- English
- Full text available
- Last 5 years range
- Meta-analyses
- Systematic reviews
- Adult (Phase 1 only)
- Human (Phase 1 only)

**Exclusion criteria [7,17]**

- Nonspinal disease
- Thoracic and lumbar disease
- Radiculopathy without myelopathy
- Other nondegenerative myelopathy
- Traumatic spinal cord injury
- Tumor/neoplasm/hemangioma/metastases
- Infection
- Arteriovenous fistula
- Radiation injuries
- Motor neuron disease/amyotrophic lateral sclerosis
- Multiple sclerosis
- Autoimmune diseases of the nervous system
- Inflammatory arthritis
- Congenital, hereditary, and neonatal diseases and abnormalities

For this illustrative search, only meta-analyses and systematic reviews were searched. This aimed to emulate the crucial initial step in performing any systematic review or meta-analysis, that is, avoiding duplication and/or identifying previous systematic reviews or meta-analyses to update. This style of search also is frequently used by clinicians to provide an efficient evidence update [18,19]. Moreover, it allowed the use of meta-analysis, systematic review, or review filters, which pragmatically reduced the labor-intensive process of hand searching. The performance of these filters is validated for identifying systematic reviews and meta-analyses, with sensitivities of up to 19.1% for meta-analyses, 22.1% for systematic reviews, and 77.5% for reviews and with specificities of up to 99.7% for meta-analyses, 99.8% for systematic reviews, and 92% for reviews [19,20].

Each search strategy phase was tested against 2 index articles identified a priori [1,21]. Of note, during the first search (*Phase 1*, searched on January 6, 2019), additional search filters (Textbox 3) were applied and trialed. However, as this *Phase 1* search strategy failed to identify assorted reference articles [1,21] prospectively collected to test the strategy, adjustments were made (Textbox 3). This refined search was termed *Phase 2* (searched on January 17, 2019).

### Evaluate Performance of the Illustrative Search Strategy

#### Databases Searched

Searching multiple databases is often required in thorough literature searches [11]. Thus, searches (Phase 2) were first carried out on the MEDLINE database via PubMed and repeated using Excerpta Medica dataBASE (EMBASE).

#### Filters (Medical Literature Analysis and Retrieval System Online)

The function of the selected common PubMed filters was also tested. Filters in Textbox 3 served as the baseline for search results. Additional filters were then added and removed, and their effects were studied. The adult, human, and English filters, common filters employed in DCM systematic reviews and meta-analyses, were examined.

#### Related Articles Function (PubMed/Medical Literature Analysis and Retrieval System Online)

The related articles feature is commonly used in research synthesis. The utility of this filter within the DCM literature base was tested on the DCM systematic review or meta-analysis results of Phase 1 (low sensitivity) and Phase 2 (high sensitivity). All of the DCM systematic review or meta-analysis articles in Phase 1 were examined. In Phase 2, a pragmatic 10% a priori of the total relevant systematic reviews or meta-analyses were hand searched. Random number table selection was used to identify these articles from our identified cohort. Our search filters (Textbox 3) were applied to each article’s related articles results, looking for additional studies not yet identified with our search strategies.

### Analysis

The outcomes measured during Phase 1 and Phase 2 searches were as follows:

1. Total number of articles returned.
2. Number of relevant articles (DCM systematic reviews or meta-analyses) meeting the inclusion criteria.
3. Categorization of irrelevant studies—using ICD-11 categories as a guide to creating themes of imprecision [22].
4. The number of additional relevant articles using the
  Related Articles
  function (MEDLINE database via PubMed).
5. The number of additional articles found using a second literature database, stratified into relevant and irrelevant articles.

## Results

### Phase 1

This search strategy returned 3439 results, refined to 175 results using the above filters and with 1 duplicate being subsequently removed. The categorization of the remaining 174 results is summarized in Multimedia Appendix 1. Of note, 18.4% (32/116) results fitted the inclusion criteria, totaled in the *DCM* category of Multimedia Appendix 1. Of these, 9.8% (17/116) DCM studies were systematic reviews or meta-analyses, with the 15 other studies consisting of case reports and narrative reviews.

With regard to the overlapping terms when considering non-DCM search results relating to the spine (116/174, 66.6% of results), the most common categories involved were spinal neoplasms (25/174, 14.4%), spinal cord injury (15/174, 8.6%), and infectious diseases of the spine and central nervous system (CNS; 15/174, 8.6%). When considering nonspinal categories (26/174, 14.9% of results), diseases of the nervous system—cerebral diseases (9/174, 5.7%)—and disorders of the urological tract and male genital tract (3/174, 1.7%) were the most commonly encountered.

As stated above, this *Phase 1* search strategy failed to identify our chosen index articles [1,21]. Resultantly, this search was refined via removal of the *adults* (>19 years old) and *human* filters and thereafter termed the *Phase 2* strategy (Textbox 3), searched on January 17, 2019.

### Phase 2

**Figure 1:**
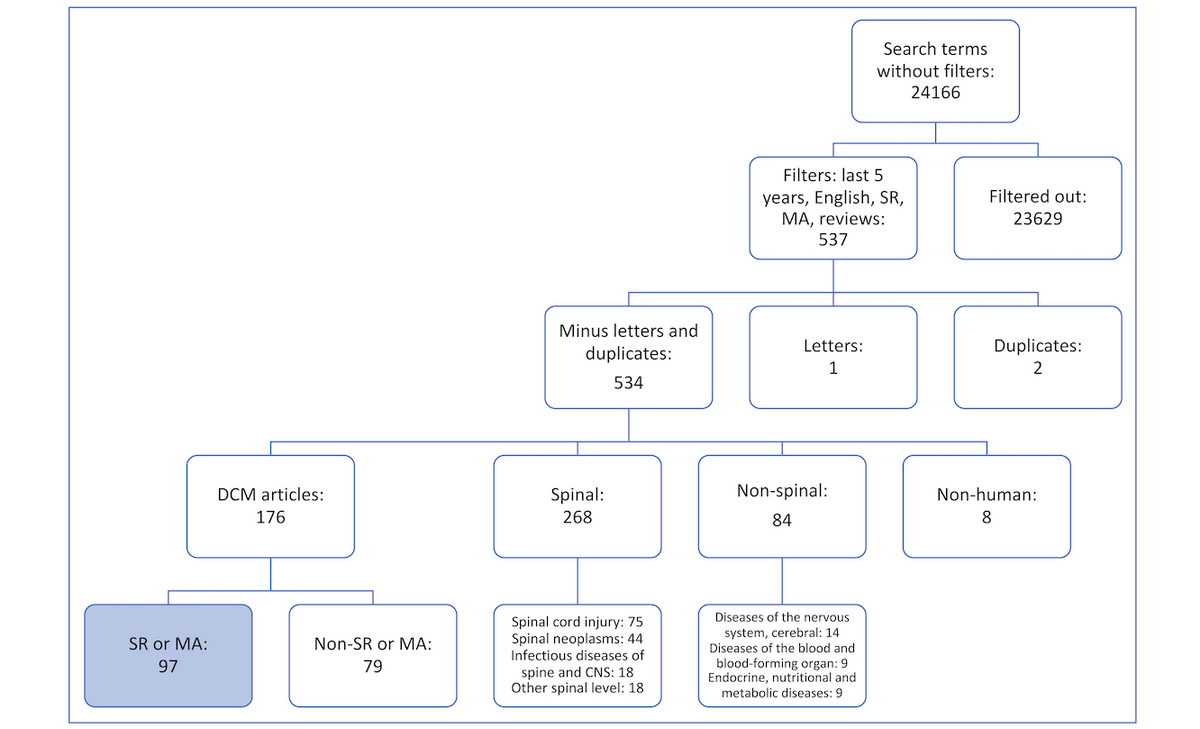
MEDLINE search strategy with the most common imprecision categories. CNS: cerebral nervous system; DCM: degenerative cervical myelopathy; MA: meta-analysis; SR: systematic review.

This search strategy returned 24,166 results, refined to 537 results using filters. Of these, 2 duplicates and 1 letter to editor publication were subsequently removed (Figure 1). The remaining 534 studies are categorized in Table 1. We found that 32.9% (176/534) of results fitted into the *DCM* category of Table 1. Of these, 18.2% (97/534) DCM studies were systematic reviews or meta-analyses, with the 79 other studies consisting predominantly of case reports and narrative reviews.

**Table 1:** Phase 2 search results categorization guided by the International Classification of Diseases 11th Revision.

Category and subcategory		Values, n (%)
**DCM^a^**		
	Systematic review or meta-analysis	97 (18.16)
	Nonsystematic review or non–meta-analysis	79 (14.79)
	Subtotal	176 (32.96)
**Non-DCM, spinal**		
	Spinal cord injury	75 (14.04)
	Spinal neoplasms	44 (8.24)
	Infectious diseases of the spine and CNS^b^	18 (3.37)
	Other spinal level (thoracic, lumbar, or sacral)	18 (3.37)
	Miscellaneous^c^	17 (3.18)
	Vascular pathologies	14 (2.62)
	Surgical techniques and complications (non-DCM)	13 (2.43)
	Traumatic spondylopathy	9 (1.69)
	Congenital spinal diseases	8 (1.50)
	Inflammatory and demyelinating diseases of the CNS	8 (1.50)
	Cervical disc disorders	7 (1.31)
	Radiology of the spine and spinal cord (non-DCM)	7 (1.31)
	Neurodegenerative disease of the CNS	7 (1.31)
	Inflammatory spondylo-arthopathies	7 (1.31)
	Cerebrospinal fluid disorders (leaks and syringomyelia)	5 (0.94)
	Deforming dorsopathies	5 (0.94)
	Metabolic diseases with spinal sequelae	4 (0.75)
	Cervical radiculopathy	2 (0.37)
	Subtotal	268 (50.19)
**Non-DCM, nonspinal**		
	Diseases of the nervous system, cerebral	14 (2.62)
	Diseases of the blood and blood-forming organs	9 (1.69)
	Endocrine, nutritional, and metabolic diseases	9 (1.69)
	Miscellaneous	8 (1.50)
	Diseases of the ear, nose, upper respiratory tract, and head and neck	7 (1.31)
	Disorders of the female genital tract	6 (1.12)
	Diseases of the musculoskeletal system and connective tissue	6 (1.12)
	Disorders of the urological tract and male genital tract	6 (1.12)
	Pain	5 (0.94)
	Diseases of the circulatory system	4 (0.75)
	Mental and behavioral disorders	4 (0.75)
	Infectious and parasitic diseases	2 (0.37)
	Diseases of the digestive system	1 (0.19)
	Diseases of the lower respiratory tract	1 (0.19)
	Subtotal	84 (15.36)
**Nonhuman**		
	Feline and Canine	6 (0.94)
	Equine	2 (0.37)
	Subtotal	8 (1.50)
Grand total		534 (100)

^a^DCM: degenerative cervical myelopathy.

^b^CNS: central nervous system.

^c^Miscellaneous: not specified in the International Classification of Diseases 11th Revision (rare genetic disorders, rare immunological disorders, and rare extrapyramidal disorders) and/or not fitting into the above categories.

Of note, our search strategy was formed from an amalgamation of strategies used by previous authors in the field and validated PubMed hedge. Resultantly, the search terms used included 2 additional terms (*DCM/Degenerative Cervical Myelopathy* and *OPLL/Ossification of Posterior Longitudinal Ligament*) to the original validated hedge by Davies et al [7]. The addition of these terms made no difference to the search results during Phase 1 or Phase 2.

#### Categories of Imprecision

In regard to the overlapping terms when considering non-DCM search results relating to the spine (268/534, 50.2% of results), the most common categories involved spinal cord injury (75/534, 13.67%), spinal neoplasms (44/534, 8.2%), infectious diseases of spine and CNS (18/534, 3.4%), and other spinal level (thoracic, lumbar, or sacral; 18/534, 3.4%). When considering nonspinal categories (8/534, 15.4% of results), diseases of the nervous system; cerebral diseases (14/534, 2.6%); diseases of the blood and blood-forming organs (9/534, 1.7%); and endocrine, nutritional, and metabolic diseases (9/534, 1.7%) were the most commonly encountered.

#### Inadequacy of PubMed Search Filters

Importantly, Phase 2, unlike Phase 1, was successful in identifying our prospectively collected reference DCM systematic review and meta-analysis articles [1,21], highlighting the unreliable nature of *human* and *adult* search filters, which were present in Phase 1.

The nonhuman category totaled to 1.5% (8/534), which does not reflect the 81 articles removed from the search results when the *human* filter is selected. Similarly, the inadequacy of the *adult* filter is demonstrated through the comparison of the relative paucity of results specific to pediatric populations. There were only 4 of these results within the result categories, compared with the removal of 358 articles on the application of this filter. In addition, the application of the English filter removed 22 articles; the distribution of these was as follows: 3 in Chinese, 4 in French, 9 in German, 4 in Japanese, 1 in Russian, and 1 in Spanish. Of these, 4 articles (all in German) were in the field of DCM but none were systematic reviews or meta-analyses. Thus, the removal of non-English articles did not decrease the sensitivity of our search. For pragmatic purposes, the *year range* and *text availability* (ie, full text availability) filters were not scrutinized.

### Extended Literature Search

#### Related Articles Function (PubMed/Medical Literature Analysis and Retrieval System Online)

The *related articles* feature was tested in 2 searches of differing sensitivities, Phase 1 (low sensitivity) and Phase 2 (high sensitivity). In Phase 1, all 32 DCM systematic review and meta-analysis articles were examined in view of the known poor sensitivity of the search without this function. A total of 3830 articles were identified by the database. Of these, 2.7% (102/3820) articles remained after applying the Phase 1 filters: humans, full text available, last 5 years range, adults (>19 years old), English, meta-analyses, systematic reviews, and reviews. These filtered studies were reviewed, and 1.8% (67/3820) of the total *related articles* search were found to be relevant to the DCM category topic. Duplicates were included in the above analysis as each article’s *related articles* were examined separately. However, after the removal of duplicates and comparison with the original 32 DCM articles, 5 relevant studies that fitted the inclusion criteria (all of which were systematic reviews or meta-analyses) were identified through this *related articles* search but were not found in the original Phase 1 search. Thus, 1 new DCM systematic review or meta-analysis was found per 6.4 articles examined. However, our selected reference articles were still not identified by this extended Phase 1 search strategy, further elucidating Phase 1’s lack of sensitivity.

In Phase 2, the *related articles* function was used on a pragmatic a priori 10% of the DCM systematic reviews and meta-analyses. This equated to 10 articles examined, chosen by a random number table. A total of 980 articles were classed as articles related to the 10 DCM systematic reviews and meta-analyses. After the application of Phase 2 filters, 118 studies remained, with 105 of these being related to DCM and 87 being DCM systematic reviews or meta-analyses. Importantly, 91% (79/87) of these DCM systematic review and meta-analysis articles were identified by the Phase 2 strategy. Thus, 7 new articles (6 once duplicates were removed) were found via this extended search, equating to 1 new DCM systematic review or meta-analysis found per 1.67 articles examined.

#### Second Database Search (Excerpta Medica dataBASE)

**Figure 2:**
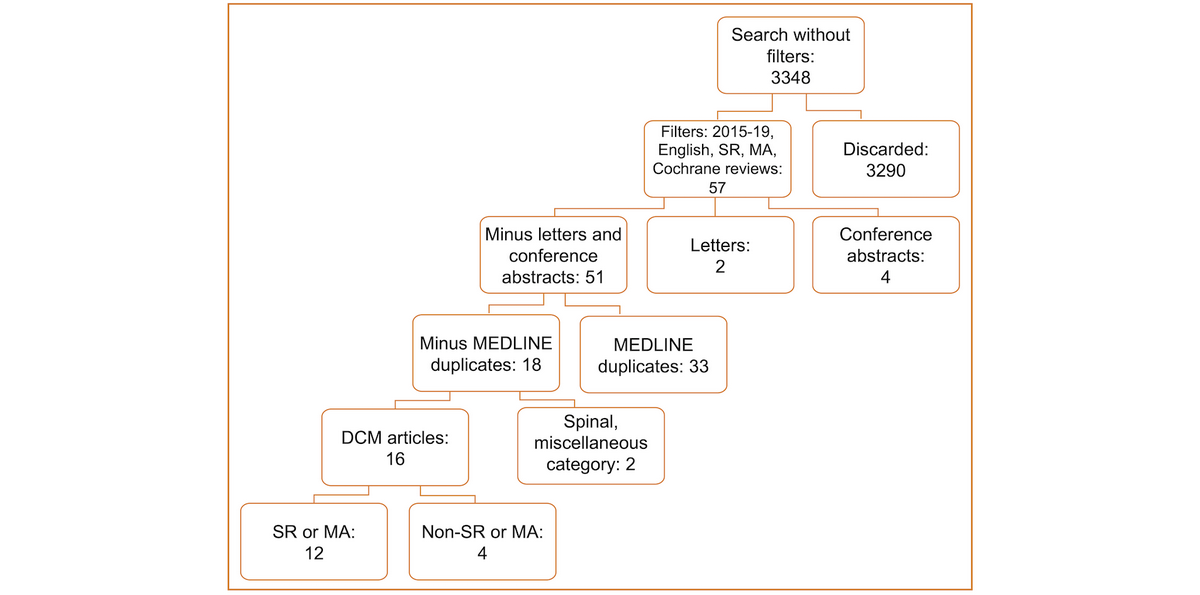
EMBASE search strategy and results. CNS: cerebral nervous system; DCM: degenerative cervical myelopathy; MA: meta-analysis; SR: systematic review.

The EMBASE database was searched on February 3, 2019, through the adaptation of the above search strategy (although originally developed for MEDLINE/PubMed) [7]. The following filters were applied to emulate the Phase 2 PubMed search: 2015-2019, humans, systematic reviews, meta-analyses, and Cochrane review (Figure 2). These filters narrowed down the raw 3348 results to 57 results; 6 nonresearch articles were removed, 51 results were compared with the Phase 2 PubMed cohort, and 33 duplicates (all within the DCM category) were subsequently removed. This left 18 articles for review, 67% (12/18) of which were systematic reviews or meta-analyses in DCM and 22% (4/18) of which were nonsystematic reviews or non–meta-analysis DCM articles.

## Discussion

### Principal Findings

Our study served to replicate an important step in research synthesis and evidence-based clinical practice, identifying systematic reviews or meta-analyses in the field. It was hypothesized that, without an agreed index term, MeSH, or an equivalent and accurate indexing of articles, this attempt at comprehensively searching the literature would be inefficient and imprecise. Specifically, only 18.2% (97/534) of search results concerned DCM systematic reviews or meta-analyses and commonly used PubMed search filters (such as *adult*) stratified studies incorrectly. Moreover, it is clear that expanding the search with *related articles* functions and searching additional databases will identify additional relevant studies. These results taken together indicate that systematic search in DCM is currently extremely labor-intensive.

The PubMed filters of *systematic reviews*, *reviews*, and *meta-analyses*, with proven satisfactory sensitivity and specificity [19,20], were applied to our search terms. The standard against which imprecision was judged was the percentage and number of DCM systematic reviews or meta-analyses found. This aimed to replicate common occurrence in clinical practice and the initial steps of research synthesis. It also served as a pragmatic approach to assessing imprecision. DCM systematic reviews and meta-analyses were 18.2% (97/534) of studies returned in our search (Phase 2), with principal categories of imprecision including spinal cord injury and spinal neoplasms.

The need to exercise caution when applying other generic filters in the field of DCM is illustrated in the results of the Phase 1 search. This included the additional filters of *adult* and *human* and a search that was less sensitive or specific than Phase 2 (searching without these filters applied). However, the addition of the English filter did not affect the number of DCM systematic reviews or meta-analyses found in our search. This is unfortunate as these filters have the potential to make this already labor-intensive process more efficient but fail to do so in DCM PubMed literature.

The *related articles* function on PubMed’s displayed utility in the setting of both low- and high-sensitivity searches. In Phase 1, it was used to find 5 additional DCM systematic review and meta-analysis studies after searching the original 32 articles—1 new DCM systematic review or meta-analysis was found per 6.4 articles examined. In Phase 2, 6 extra articles were found via this extended search, equating to 1 new DCM systematic review or meta-analysis found per 1.67 articles examined. Thus, the common practice of using this function for the literature search is justified and recommended. In addition, it is important to note that the EMBASE database composed 14% (16/114) of the total EMBASE plus MEDLINE *DCM systematic reviews* *and meta-analysis* yield of our search and 9.2% (18/195) of the total EMBASE plus MEDLINE *DCM* category results. This reiterates the potential value of searching multiple literature databases while performing systematic reviews or meta-analyses in the field of DCM, and DCM reviewers should be cognoscente of this [11].

### Study Results in Context

The findings of our search reflect previous studies discussing systematic review or meta-analysis retrieval in other fields. Specifically, they mirror the search precision generated by a DCM hedge. Overlapping terms and general imprecision have spurred the creation of search hedges, aiming to increase search efficiency when performing comprehensive literature retrieval [23]. However, there is a broad range of sensitivities and specifies that is achieved using these hedges [23,24]. This is compounded by the inaccuracy of generic search filters, for example, the *cross-sectional studies* filter [25]. There is an agreement that root problems to such search inefficiencies included interindexer inconsistency when labeling studies and a lack of natural language processing terms such as MeSH terms [26,27]. Regardless, searching of multiple databases and using *related articles*-type functions are widely accepted for their utility [28]. Importantly, the choice of databases must be carefully considered. For example, Google Scholar is another option commonly considered for literature retrieval. It holds advantages in its simplicity, familiarity, and ability to search a broader area of the literature (including multiple medical libraries and preprint articles) [29]. However, it has been criticized for being less comprehensive, less precise, and less sophisticated (in terms of advanced search functions and controlled vocabulary) [29,30]. Therefore, we elected dedicated literature search databases for the purposes of identifying imprecision.

### Developing a Solution

As the rate of our primary research synthesis exceeds our ability to review it [31], it is imperative that our methods for systematically reviewing and analyzing data emphasize efficiency. Over the last decade, an average of 700,000 to 850,000 articles per year were published in MEDLINE [32], whereas 2500 systematic reviews are published yearly [12]. It is estimated that 10,000 Cochrane systematic reviews would be needed to sufficiently synthesize the information from 300,000 trials in the Cochrane Central Register of Controlled Trials literature database [33]. This was thought by Cochrane to be achievable by 2010 to 2015, but to date, this figure stands at approximately 7900 [33]. Furthermore, although Cochrane aspires to update these reviews regularly with new studies and analyses, it struggles to do so [12]. The suggested reason for this is the inefficiency of the systematic review or meta-analysis process [2]. Proposed methods to ameliorate this issue include standardization (eg, Preferred Reporting Items for Systematic Reviews and Meta-Analyses and International Prospective Register of Systematic Reviews) and availing of technology to streamline the process [15].

Although technology such as meta-search engines, machine learning platforms, and automated information extraction systems continue to develop, these solutions remain largely experimental [34-37]. Filter or hedge development has been proposed as one solution to this problem. However, as demonstrated in this study, this can have varying degrees of accuracy. When developing a DCM search filter with 100% sensitivity, this returned <20% precision values, and efforts to optimize its specificity using *NOT* functions reduced the sensitivity [7].

A more comprehensive change would be the development of an index term/ICD category with a paired MeSH term. This could deliver immediate search efficiencies. MeSH terms have been developed as natural language processing tools [38], streamlining the current literature search process, and will likely prove integral to a future machine-assisted and/or machine-led review of the literature [37]. Indeed, MeSH tags have the potential to solve the identified issues in DCM literature of heterogenous synonyms and overlapping terms with non-DCM literature. The hierarchal tree structuring of MeSH tags will allow encompassing the various index terms that exist for DCM without the inclusion of such a large body of the literature, which is unrelated but shares isolated overlapping words or phrases. The MeSH labeling process, once a MeSH term is created, has moved from a human-only process to a machine-assisted process, saving cost and time for literature libraries [27]. Each article is currently processed by using the Medical Text Indexer technology, suggesting MeSH labels (on the basis of the title, abstract, and related articles’ labels) to human indexers [27]. However, the rate of comprehensive and accurate indexing struggles to keep up with high rates of research synthesis [27]. However, novel fully automatic MeSH indexing technologies (eg, MeSHLabeler and DeepMeSH) employ machine learning algorithms to make large-scale MeSH indexing cheaper, more efficient, and more accurate. Employing such technology should motivate us to aim for a fully indexed body of DCM literature [27,39].

Index terms are equally important in standardizing our language in both research and clinical practice. In addition to search inefficiencies, inconsistencies within the definition of DCM have prevented all retrieved studies being pooled for analysis [40]. Development of a universally agreed definition has been successfully done via consensus processes for other diseases, more specifically via a modified Delphi process [41]. Our group aims to establish this index term for DCM as part of our Research objectives and Common Data Elements for DCM study. For creation of an ICD entry, a proposal can be made via the World Health Organization web-based ICD-11 platform to be reviewed by a Topic Advisory Group and Revision Steering Group [42].

### Limitations

This illustrative search excluded the vast majority of primary DCM research by using the systematic review, review, and meta-analysis filters. This was done to make the illustration of imprecision, a process that requires hand searching of articles, more pragmatic. Although this reduced the number of articles retrieved, given that the objective of this study was to consider the sources of imprecision, we do not feel that this would have limited our findings. Moreover, the practice of limiting research synthesis is reflective of day-to-day search practices.

In addition, a small number of results covered multiple categories of imprecision. In these cases, a review of the article’s full text for the primary area of discussion was undertaken, followed by allocation to that imprecision category. We acknowledge that this is a relatively subjective process. However, imprecision because of overlapping terms was still identified, regardless of categorization, and thus, the primary aims of the study were fulfilled. Finally, we searched only 1 additional database, and only 10% of articles had their related articles function tested. Again, this served to elucidate their known utility in a practical fashion, with further database evaluation not required.

### Conclusions

This paper illustrates the difficulties encountered by past, current, and future reviewers of DCM literature. Overlapping and heterogenous search terms and inaccurate article indexing lead to an imprecise and wasteful process. Researchers in the field of DCM must be aware of the adverse effects that sensitivity and specificity common search functions (eg, *humans* and *adult*) may have on the retrieval of results. However, the common practice of using *related articles* functions and searching multiple databases is recommended in DCM literature. Looking forward, MeSH labeling, a standardized DCM definition, and comprehensive indexing of DCM literature will be crucial steps in ameliorating these hurdles.

## Appendix

Multimedia Appendix 1 Phase 1 Search Results Categorisation.

## Abbreviations

CNS: central nervous system
CSF: cerebrospinal fluid
DCM: degenerative cervical myelopathy
EMBASE: Excerpta Medica dataBASE
ICD-11: International Classification of Diseases 11th Revision
MEDLINE: Medical Literature Analysis and Retrieval System Online
MeSH: Medical Subject Headings
