# Supplementary material for: Tackling Research Inefficiency in Degenerative Cervical Myelopathy: Illustrative Review
Source: JMIR Res Protoc. 2020 Jun 11;9(6):e15922. doi: 10.2196/15922 (PMC7317636; doi:10.2196/15922)
Supplement: Multimedia Appendix 1 [file resprot_v9i6e15922_app1.docx]

## Appendix:

Appendix 1. Phase 1 Search Results Categorisation. SR = systematic review, MA = meta-analysis, DCM = degenerative cervical myelopathy, CNS = cerebral nervous system. CSF = cerebrospinal fluid.

| **Category** | **Sub-Category** | **Number** | **Percentage** |
| --- | --- | --- | --- |
| *DCM* |  |  |  |
|  | SR or MA | 17 | 9.77% |
|  | Non-SR or MA | 15 | 8.62% |
|  | Subtotal | 32 | 18.39% |
|  |  |  |  |
| Non-DCM,  Spinal |  |  |  |
|  | Congenital spinal diseases | 3 | 1.72% |
|  | CSF disorders (leaks and syringomyelia | 5 | 2.87% |
|  | Infectious diseases of the spine and CNS | 15 | 8.62% |
|  | Radiology of the spine & spinal cord (non-DCM) | 2 | 1.15% |
|  | Inflammatory & demyelinating diseases of the CNS | 6 | 3.45% |
|  | Other spinal level (thoracic, lumbar, sacral) | 9 | 5.17% |
|  | Inflammatory spondylo-arthopathies | 3 | 1.72% |
|  | Spinal cord injury | 15 | 8.62% |
|  | Surgical techniques & complications (non-DCM) | 4 | 2.30% |
|  | Traumatic spondylopathy | 5 | 2.87% |
|  | Spinal neoplasms | 25 | 14.37% |
|  | Vascular pathologies | 11 | 6.32% |
|  | Cervical disc disorders | 3 | 1.72% |
|  | Metabolic diseases with spinal sequelae | 2 | 1.15% |
|  | Miscellaneous^a^ | 8 | 4.60% |
|  | Subtotal | 116 | 66.67% |
|  |  |  |  |
| *Non-spinal* |  |  |  |
|  | Diseases of the nervous system, cerebral | 9 | 5.17% |
|  | Diseases of the ear, nose, upper respiratory tract, head & neck | 2 | 1.15% |
|  | Disorders of the female genital tract | 2 | 1.15% |
|  | Diseases of the blood and blood-forming organ | 2 | 1.15% |
|  | Infectious and parasitic diseases | 2 | 1.15% |
|  | Diseases of the musculoskeletal MSK system and connective tissue | 2 | 1.15% |
|  | Pain | 1 | 0.57% |
|  | Mental and behavioural disorders | 1 | 0.57% |
|  | Disorders of the urological tract and male genital tract | 3 | 1.72% |
|  | Diseases of the circulatory system | 1 | 0.57% |
|  | Miscellaneous*^a^* | 1 | 0.57% |
|  | Subtotal | 26 | 14.94% |
|  |  |  |  |
| **Total** |  | **174** | **100.00%** |

^a^ Miscellaneous - Assorted subjects not fitting into above general categories
